# Into the fold: advances in understanding aPKC membrane dynamics

**DOI:** 10.1042/BCJ20230390

**Published:** 2023-12-15

**Authors:** Mathias Cobbaut, Peter J. Parker, Neil Q. McDonald

**Affiliations:** 1 Signalling and Structural Biology Laboratory, The Francis Crick Institute, NW1 1AT London, U.K.; 2 Protein Phosphorylation Laboratory, The Francis Crick Institute, NW1 1AT London, U.K.; 3 School of Cancer and Pharmaceutical Sciences, King's College London, London, U.K.; 4 Department of Biological Sciences, Institute of Structural and Molecular Biology, Birkbeck College, London, U.K.

**Keywords:** apkc, atypical pkc, cell polarity, phospholipids, protein kinase c

## Abstract

Atypical protein kinase Cs (aPKCs) are part of the PKC family of protein kinases and are atypical because they don't respond to the canonical PKC activators diacylglycerol (DAG) and Ca^2+^. They are central to the organization of polarized cells and are deregulated in several cancers. aPKC recruitment to the plasma membrane compartment is crucial to their encounter with substrates associated with polarizing functions. However, in contrast with other PKCs, the mechanism by which atypical PKCs are recruited there has remained elusive until recently. Here, we bring aPKC into the fold, summarizing recent reports on the direct recruitment of aPKC to membranes, providing insight into seemingly discrepant findings and integrating them with existing literature.

Atypical PKCs (aPKCs) are serine/threonine protein kinases belonging to the protein kinase C family. In contrast with classical and novel PKCs they do not contain a C2 domain and DAG-responsive C1 domains. Instead, the regulatory module (RM) N-terminal of their kinase domains contains a Phox and Bem1 (PB1) domain, pseudo-substrate sequence (PSS) and DAG-insensitive C1 domain (Figure 1A). In humans two isoforms are present (aPKCι/ζ) that share ∼73% sequence identity. Despite their high homology and overlapping functions, they also have context-specific biological functions as well as differential and sometimes opposing effects in cancer [1–3].

**Figure 1.**
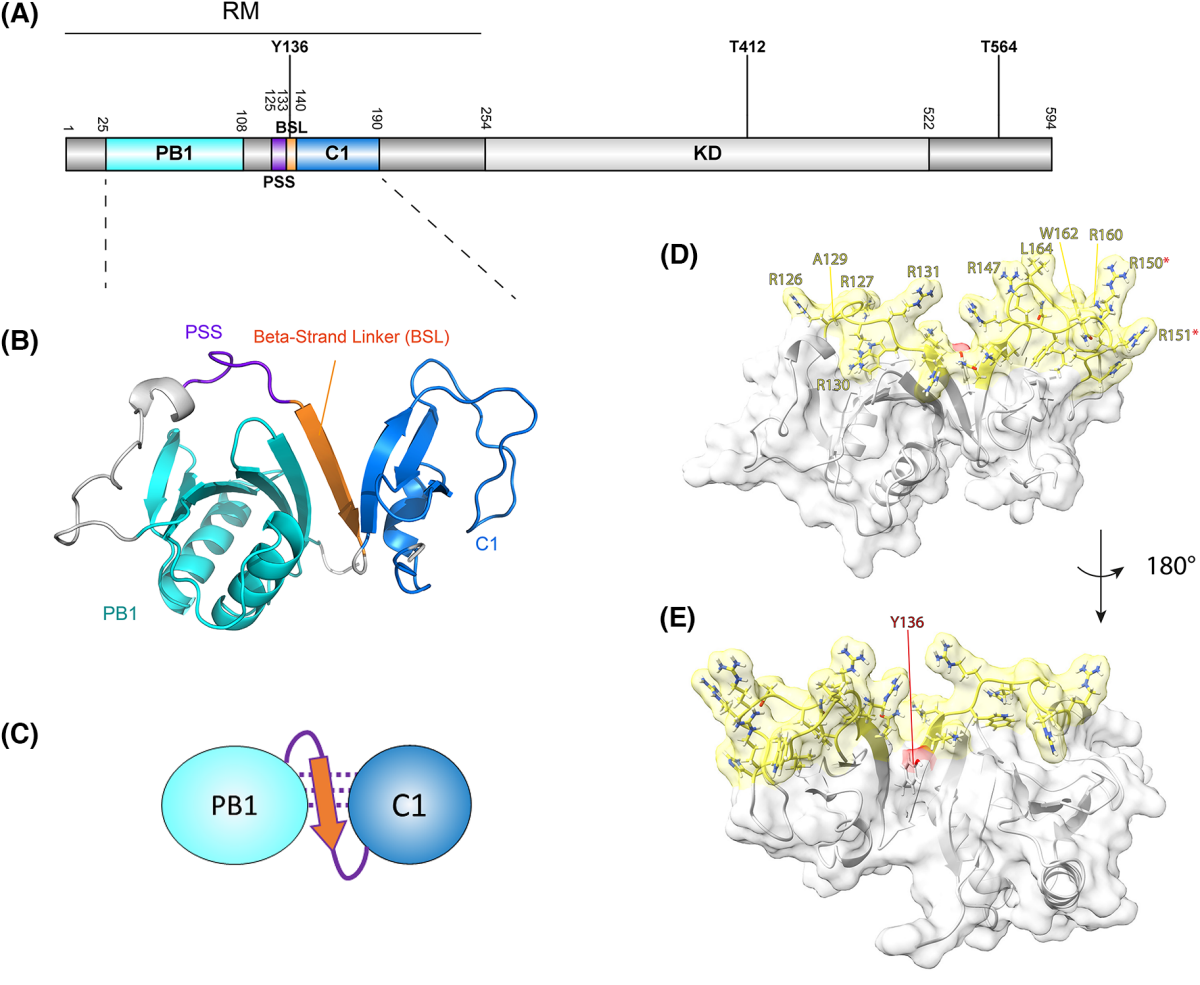
The aPKC N-terminal domains are predicted to be organized into a single functional module, the regulatory module (RM), with membrane binding determinants oriented in a single continuous surface. (**A**) Representation of the primary structure of aPKC (human aPKCι residue numbering) with protein domains and the three most abundantly detected phosphorylation sites indicated (cf. PhosphoSitePlus database). RM, regulatory module; PB1, Phox and Bem1 domain; PSS, Pseudo-substrate sequence; BSL, beta-strand linker; KD, kinase domain; T412, activation loop residue; T564, turn motif residue. (**B**) AlphaFold2 colab prediction of the aPKC^RM^, with the PB1 domain (cyan), PSS (purple) linked via a Beta-Strand Linker (orange) to the C1 domain (blue). Figure generated in PyMol. (**C**) Schematic representation of **B**. (**D**) Surface representation of the RM with the predicted lipid-binding residues within the RM color coded in yellow. Residues involved in membrane-association as identified in [11] are labeled. Arg150/151 are part of a NLS as identified in [21] and denoted with a red asterisk. Figure generated in ChimeraX. (**E**) Indication of the Tyr-136 residue location, which in the unphosphorylated state is part of the BSL. Figure panels B–E are adapted from [11].

aPKCs are central to the establishment and maintenance of cell polarity in a variety of tissues and across many eukaryotic species [4,5]. Establishing and maintaining apico-basal polarity in epithelia or asymmetrically dividing cells, are intricate processes characterized by distinct plasma membrane compartments with unique identities wherein specific protein complexes are assembled to orchestrate domain identity. In the apical domain this includes Par proteins and crucially aPKC. However, how aPKC is recruited to and spatially organized within this compartment has been poorly understood. Indirect recruitment has been proposed through interaction with protein partners such as Par-3 and Cdc42/Par-6 [6–9], but whether aPKC can bind membranes directly in cells has remained largely elusive. This critical question has been in the spotlight recently with several groups reporting apparently conflicting evidence for the involvement of different aPKC regions in membrane recruitment [10–12]. The regions within the RM (i.e. outside the kinase domain) proposed to engage lipids are the C1 domain and PSS motif (Figure 1A). Here, we provide an overview of these recent findings and integrate them into a comprehensive picture.

Dong et al. [10] showed that in HEK293 cells, MDCK cells and the *Drosophila* follicular epithelium, the PSS is crucial for aPKC membrane targeting. They further showed the PSS region interacts with the phosphorylated phosphoinositides (PIs) PtdIns4P and PtdIns4,5P_2_ in cells, by studying the effect of rapamycin-inducible PI phosphatases on aPKCζ membrane recruitment. This PI-dependent recruitment is driven by the PSS as shown by liposome pelleting assays using WT and PSS-charge disrupting mutants of aPKCζ. The responsiveness of the PSS to PIs is corroborated by a recent *in vitro* study showing that phosphatidylinositol 3-phosphate (PtdIns3P) and PtdIns4P binding to aPKCι is strongly dependent on the PSS region [13]. The PSS region (and not the C1 domain) in aPKCι was also implicated in binding PtdIns3,4,5P_3_ by an earlier study, where it could activate the kinase downstream of insulin stimulation [14]. This is in line with another study by Nakanishi et al. [15] showing a stimulatory effect of PtdIns3,4,5P_3_ on aPKCζ activity in conjunction with PtdSer and their combined requirement for autophosphorylation. However, other studies indicated that PI effects on aPKCζ showed little distinction between PtdIns3,4,5P_3_ and its precursor PtdIns4,5P_2_ [16]. While the majority of evidence points towards PIs being involved in PSS binding, we note that PtdSer-mediated activation of aPKCζ has also been shown to be driven in part by the PSS in a deletion mutagenesis study looking at kinase activation, and that the Newton laboratory showed that in a mixed-micelle context PIs do not activate aPKCζ, whereas PtdSer does [17,18]. The overall observation that the aPKC PSS can interact with PIs agrees with the long-standing evidence that PSS peptides can mediate interactions with anionic phospholipids [19].

A separate study by Jones et al. [12] reported recently that membrane binding is dependent predominantly on the C1 domain in mitotic *Drosophila* neuroblasts and the larval brain inner proliferation center (IPC) epithelium, where PSS deletions have little effect. That the isolated aPKCι/ζ C1 domains can be recruited to the membrane compartment was previously also shown by the Blumberg laboratory in LNCaP cells [20]. Recruitment in interphase cells is quite inefficient however and the C1 domain is mainly nuclear, similar to what has been observed for the RM in interphase HEK293 cells [11,20]. This is likely due to a nuclear localization signal (NLS) present in one of the C1 loops (Figure 1D) [21]. The C1 domain in atypical PKCs does not respond to diacylglycerol (DAG) unlike classical and novel PKCs, because it has accumulated basic residues in place of the hydrophobic positions important for DAG interaction [20,22]. However, the C1 domain can bind directly to anionic phospholipids such as phosphatidylserine (PtdSer), phosphatidylglycerol (PtdGro) and unconjugated phosphatidic acid [12]. This is in line with previous observations that aPKCζ can bind and is activated by PtdSer [15,18]. This lipid specificity is similar to the C1b domain in classical and novel PKCs, which has also been shown to bind PtdSer and other anionic phospholipids [23]. Recent structures of the PKCδ C1b domain with ligands bound show how phospholipids pack against the periphery of the C1b loops with their phosphate headgroups [24]. These phospholipid binding sites correspond to the predicted membrane-binding regions mutagenized in recent studies on aPKCι but whether this precise binding arrangement is also true for the atypical PKCs that lack a DAG binding site remains to be determined [11].

In a third study we demonstrated that both the C1 and PSS can act in concert to promote membrane binding, and this in principle offers some resolution to the C1 versus PSS debate [11]. The basis for this conclusion derives from structure prediction which indicates that predicted membrane-binding residues within the C1 domain and PSS form a contiguous arrangement in the isolated (i.e. kinase domain disengaged) aPKCι RM (Figure 1B–E) [11]. Mutation of predicted membrane-binding residues in either the C1 domain or PSS results in a loss of membrane binding in mitotic cells (Figure 1D). These observations are in line with deletion mutagenesis studies demonstrating lipid activation *in vitro* requires both the PSS and C1 domain [17]. The coupled organization of membrane binding determinants is fostered by the orientation of the PB1 domain and its tethering to the C1 domain by a β-strand linker (BSL) motif. This short β-strand comprising residues 133–138 in aPKCι (RKLYCA) is predicted to bridge and stabilize the PB1 and C1 domains and to ensure the positioning of the membrane-binding residues [11]. Crucially the BSL can be phosphorylated and a phospho-mimetic substitution causes disruption, uncoupling PB1 and C1 domains and the continuous arrangement of the RM, resulting in a reduction in membrane binding propensity [11]. The BSL sequence is highly conserved in evolution. For example, a sequence alignment containing this motif as output of a CDART analysis (https://www.ncbi.nlm.nih.gov/Structure/lexington/lexington.cgi) reveals a 97% sequence conservation of the Tyr residue [25]. A His substitution seems to be tolerated in the motif's capability to form the BSL, but substitutions with polar residues result in a predicted loss of this structure (Table 1). Given that the C1 domain interacts predominantly with PtdSer and the PSS with PIs, suggests a mixed lipid environment is likely the most conducive for aPKC recruitment.

**Table 1:** Sequences flanking the PSS motif and their propensity to form a ß-strand linker (BSL) as predicted by AlphaFold2 Colab

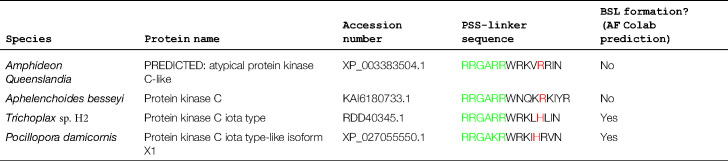

The PSS sequence is indicated in green, the substituted Tyr136 (human aPKCι) position is indicated in red.

The existence of a bi-partite lipid-binding PSS-C1 module within aPKC that targets membranes does not preclude one or other domain within the module acting in a dominant manner in a given cellular context, where for example the affinity/avidity of one motif was sufficient to dictate the steady state distribution of the protein. One possible reason for the observed dominance of the C1 domain for membrane targeting in neuronal tissues might be the relatively high abundance of PtdSer. Nearly 15% of lipids in the brain are PtdSer, which is in higher abundance than in other tissues such as liver (3.1%) and the adrenal gland (2.5%) (rat) [26]. It is therefore conceivable that the avidity of aPKC for membrane binding can be reached solely by C1 engagement in neuronal cells, whereas in other tissues a dependency on PIs may be more pronounced (Figure 2A).

**Figure 2.**
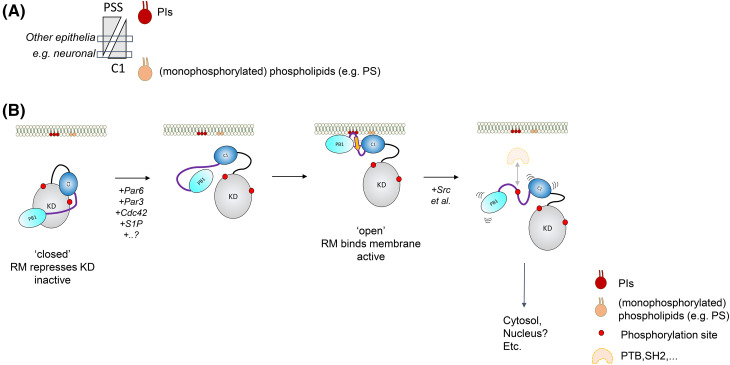
Lipid and protein factors that mediate membrane binding and release. (**A**) Representation of the lipid binding properties and dependencies on membrane binding regions within the aPKC^RM^. The PSS predominantly binds PIs, while the C1 binds PtdSer, PtdGro and other anionic lipids. In neuronal tissues the abundance of the latter likely provides the affinity for the RM to bind via the C1 domain, whereas in other epithelia a combined PI-dependency is likely. (**B**) Model detailing the conformational states of cytosolic and membrane bound forms of aPKC. Cytosolic and monomeric aPKC exists in an autoinhibited conformation with the PSS and C1 domain engaging the kinase domain. Upon binding of the indicated protein and lipid factors, this inhibitory module becomes exposed and undergoes a conformational change to establish a more compact RM, in which an interdomain BSL (orange) is formed to couple the PB1 and C1 domains; this organizes a rigid membrane binding platform containing residues from both the PSS and C1 domain. Phosphorylation at Tyr-136 in aPKCι driven by Src (and potentially other kinases) results in a loss of the BSL and the integrated membrane platform and consequently membrane affinity. The resulting cytoplasmic kinase is in a more open conformation and potentially subject to pY binding protein modules that may support directed activity. For more details see text.

Differences between isoforms can also account for differences in membrane binding. While comparative data are sparse, an *in vitro* study using lipid overlay assays identified differences between iota and zeta isoforms [13]. While aPKCζ bound PtdSer and phosphatidic acid in these assays, aPKCι additionally bound monophosphorylated PIs. Remarkably, both the kinase domain of aPKCι/ζ were found to bind these PIs, despite the overall acidic isoelectric point of their kinase domains. The binding site for these lipids in the kinase domain was proposed to be the docking site for the C1 domain. While this is unlikely due to the acidity of this patch in the N-lobe as identified by Zhang et al. [27], the di-basic kinase RIPR motif in the C-lobe, which also drives substrate interaction and is found mutated in cancers may in principle accommodate such phospholipids [3,28,29]. In cellular models however the PSS and C1 dominate the membrane-binding interaction for *Drosophila* aPKC and human aPKCζ (cf. above) [10,12]. The fact that the full length-aPKCι preparations display binding to PIs in these *in vitro* assays can potentially be attributed to the fact that it has a less tightly closed conformation than aPKCζ, resulting in the RM and especially the PSS being more exposed, an event that would be under more stringent control of Par6 binding in aPKCζ, which can also intrinsically bind PIs to its PSS in the RM as noted above [10]. This difference in isoforms is further corroborated by the fact that the aPKCζ construct employed has very low basal activity in kinase assays compared with aPKCι [13].

With both discrete membrane-binding regions of aPKC identified and their coupling within the RM established, efforts can be focussed on the mechanisms that promote exposure of these motifs, as well as the mechanisms regulating the lifetime of membrane occupation. When monomeric, aPKCs likely reside in a closed conformation in the cytoplasm, with both C1 and PSS engaged with the kinase domain (KD) and repressing activity (Figure 2B) [17,27]. Based on HDX-MS studies by Zhang et al. [27], the RM is likely discontinuous, to allow the PSS to engage with the active site and substrate cleft. The current AlphaFold model (version 01/11/2022) for human fl-aPKCι/ζ predicts the RM to be in a compact arrangement with high confidence, but with a high predicted alignment error (PAE) for the contacts between the RM and KD [30]. This prediction is likely weighted to the formation of these individual domains, and may furthermore not reflect the nucleotide-bound, primed state of the kinase. When predicting a ΔPB1 mutant of the kinase using AlphaFold2 Colab to reduce the constraints on the formation of the single-fold RM, the PSS docks in the substrate binding cleft in a substrate-binding mode with low PAE scores vis-a-vis its KD positioning and the C1 domain position is consistent with the studies in Zhang et al. [27]. The RM may therefore exist in an extended arrangement with the PB1 and C1 domains held apart by the docking interaction with the kinase domain, preventing non-specific uncoordinated membrane binding (Figure 2B). It is predicted that Par-6 binding would increase the extent of the open (i.e. RM disengaged from the KD) versus the closed (engaged with the KD) state, promoting membrane binding [10,31]. Par-6 may additionally help stabilize aPKC at the membrane by interacting with Cdc42 through the semi-CRIB motif. Par-3 may also expose the RM for membrane binding by interacting with the aPKC C-tail [12]. Lipids that bind the kinase domain directly have also been implicated in promoting the open conformer. Sphingosine 1-phosphate (S1P) has been modeled to bind to a basic pocket close to the substrate binding site in PKCζ, likely promoting PSS release and activation. This is corroborated by the finding that micelles containing S1P increased aPKCζ sensitivity to PtdSer induced activation [32]. Ceramide also has been shown to activate aPKCζ and its binding site has been mapped to a 20 kDa C-terminal fragment of aPKCζ comprising part of the C-lobe and C-tail [33,34]. The exact mechanism for direct activation of the enzyme remains unclear however. Regarding the context-dependent membrane interactions noted above, it is likely that there are broadly three elements to membrane recruitment at play: C1, PSS and protein partners (e.g. Par-6/Cdc42 interaction through the PB1 domain) (Figure 2B). The conformational interplay between these elements is no doubt critical to recruitment, retention and release.

Of note, all membrane-bound complexes are likely to maintain aPKC in the open/active conformation, as the domains employed in membrane targeting are important in the repression of kinase activity through direct engagement with the kinase domain (Figure 2B) [17,31]. As aPKC isozymes are primed after synthesis with activation loop and turn-motif phosphorylation, a substantial pool of aPKC kinase domains are likely retained in an active conformation upon lipid binding [18,35,36]. Once engaged in specific lipid environments, kinase activity can still be further controlled by substrate access and phosphatase activities. Unlike many kinases, aPKC not only forms transient complexes with its substrates but a subset are maintained in stable complexes such as with Lgl and Par-3 [37,38]. This is due, in part, to a docking motif flanking the phospho-acceptor site of many aPKC polarity substrates, which contributes to their high affinity for the kinase domain [37]. Such stable interactions may prevent aPKC activity towards other substrates by sterically occluding the active site, equally other docking site motifs have also been identified distal to the substrate binding cleft [29]. Phosphatase activity targeting the activation loop site in the open conformer may also influence the activated state of the kinase. For example, PP2A has been shown to colocalize with aPKCζ and negatively regulate its role in tight junction assembly in MDCK cells [39]. It is noted that the activation loop dephosphorylated aPKCι has ∼10% of the activity of the phosphorylated form [40].

aPKC's function at the membrane can be negatively regulated by mechanisms that actively promote membrane dissociation. Recent evidence suggest post-translational modification of aPKC, in particular phosphorylation within the BSL motif, can contribute to membrane release (Figures 1A,E and 2B) [11]. Phosphorylation of Tyr-136 in the aPKCι RM is only observed in the cytosolic fraction of mitotic cells and the phospho-mimetic mutation Tyr136 > Glu inhibits membrane recruitment. Whether the Tyr-136 phosphorylated form of aPKCι, displaying modestly increased basal activity, has any cytosolic function is yet to be determined (it is possible dissociation occurs with other partner proteins, including those that may dock to the pY136 peptide, impacting aPKC cytosolic conformation; Figure 2B). It also remains to be determined whether Tyr-136 phosphorylation also occurs in aPKCζ as this event has only been identified for aPKCι in mass-spec studies [41]. However, this could be due to the fact that tryptic fragments seeking to identify Tyr phosphorylation in aPKCζ will be very short due to the presence of an Arg C-terminal to this residue (compared with a Cys in aPKCι) and unlikely to be readily observed. Of note, kinase activity itself regulates membrane residency time as well. Inhibiting or mutating aPKC to a kinase-dead form results in uniform (i.e. unpolarized) membrane association [12,42]. Several mechanisms could be at play here, including *cis*-autophosphorylation, the phosphorylation of an associated partner protein, as recently proposed for Cdc42 [43], or an allosteric mechanism exposing the C1 domain [12,27]. In addition, the compartment in which dissociation occurs — i.e. the plasma membrane, or in an intracellular compartment arising from the initial (apical) plasma membrane recruitment should also be considered.

It is also of interest to consider the competing processes involved in subcellular localization. As noted above there is evidence of aPKC C1 domains being recruited to the nucleus in interphase cells. There is also an abundance of evidence on intact aPKC recruitment to nuclei, perhaps like PKCδ driven by additional NLS motifs [44,45]. What form of the kinase is the primary target imported into the nucleus; do competing influences balance the net distribution of aPKC or are there other events that drive one or other effect? There is for example evidence for aPKC being nuclear in poor prognosis tumors and several nuclear targets of aPKC have been described in these contexts [46,47]. This could be correlated with an oncogenic gain of tyrosine kinase activity towards Tyr-136 that triggers loss of the protein from membranes and a net accumulation in the nucleus, an event that has previously been associated with aPKC phosphorylation at Tyr-256 [48]. It is interesting to speculate that if aPKCι and not PKCζ were sensitive to this event this could be correlated with differences between isoforms with different and sometimes opposing roles in cancer initiation and progression [1,3].

Future research in this complex interplay between phospholipids, aPKC and its associated proteins, will help elucidate the underlying molecular mechanisms that determine the establishment and dissolution of polarized states. Integrated into this, we need to understand how regulatory events impact the default behavior. This includes where and when receptor/cytosolic tyrosine kinase(s) regulate aPKC membrane binding and how this or other pathways are tied into the shift to nuclear localization which has pathological consequences. For now, these new insights into membrane binding and release properties of the atypical isoforms complement the PKC family membrane binding puzzle and provide a clearer template on which to elucidate these broader issues.

## Open Access

Open access for this article was enabled by the participation of The Francis Crick Institute in an all-inclusive *Read & Publish* agreement with Portland Press and the Biochemical Society under a transformative agreement with JISC.

## Abbreviations

BSL: β-strand linker
DAG: diacylglycerol
KD: kinase domain
NLS: nuclear localization signal
PAE: predicted alignment error
PB1: Phox and Bem1
PIs: phosphoinositides
PSS: pseudo-substrate sequence
RM: regulatory module

## References

[1] Moscat, J., Linares, J.F., Duran, A. and Diaz-Meco, M.T. (2022) Protein kinase Cλ/ι in cancer: a contextual balance of time and signals. Trends Cell Biol. 32, 1023–1034 10.1016/j.tcb.2022.04.002 35501226 PMC9716658

[2] Reina-Campos, M., Diaz-Meco, M.T. and Moscat, J. (2019) The dual roles of the atypical protein kinase Cs in cancer. Cancer Cell 36, 218–235 10.1016/j.ccell.2019.07.010 31474570 PMC6751000

[3] Parker, P.J., Brown, S.J., Calleja, V., Chakravarty, P., Cobbaut, M., Linch, M. et al. (2021) Equivocal, explicit and emergent actions of PKC isoforms in cancer. Nat. Rev. Cancer 21, 51–63 10.1038/s41568-020-00310-4 33177705

[4] Hong, Y. (2018) aPKC: the kinase that phosphorylates cell polarity. F1000Res. 7, F1000 Faculty Rev-903 10.12688/f1000research.14427.1 PMC6020718 29983916

[5] Buckley, C.E. and St Johnston, D. (2022) Apical–basal polarity and the control of epithelial form and function. Nat. Rev. Mol. Cell Biol. 23, 559–577 10.1038/s41580-022-00465-y 35440694

[6] Wodarz, A., Ramrath, A., Grimm, A. and Knust, E. (2000) Drosophila atypical protein kinase C associates with Bazooka and controls polarity of epithelia and neuroblasts. J. Cell Biol. 150, 1361–1374 10.1083/jcb.150.6.1361 10995441 PMC2150710

[7] Franz, A. and Riechmann, V. (2010) Stepwise polarisation of the Drosophila follicular epithelium. Dev. Biol. 338, 136–147 10.1016/j.ydbio.2009.11.027 19962374

[8] Wang, S.-C., Low, T.Y.F., Nishimura, Y., Gole, L., Yu, W. and Motegi, F. (2017) Cortical forces and CDC-42 control clustering of PAR proteins for *Caenorhabditis elegans* embryonic polarization. Nat. Cell Biol. 19, 988–995 10.1038/ncb3577 28737772

[9] Martin-Belmonte, F., Gassama, A., Datta, A., Yu, W., Rescher, U., Gerke, V. et al. (2007) PTEN-mediated apical segregation of phosphoinositides controls epithelial morphogenesis through Cdc42. Cell 128, 383–397 10.1016/j.cell.2006.11.051 17254974 PMC1865103

[10] Dong, W., Lu, J., Zhang, X., Wu, Y., Lettieri, K., Hammond, G.R. et al. (2020) A polybasic domain in aPKC mediates Par6-dependent control of membrane targeting and kinase activity. J. Cell Biol. 219, e201903031 10.1083/jcb.201903031 32580209 PMC7337507

[11] Cobbaut, M., McDonald, N.Q. and Parker, P.J. (2023) Control of atypical PKCι membrane dissociation by tyrosine phosphorylation within a PB1-C1 interdomain interface. J. Biol. Chem. 299, 104847 10.1016/j.jbc.2023.104847 37211093 PMC10333572

[12] Jones, K.A., Drummond, M.L., Penkert, R.R. and Prehoda, K.E. (2023) Cooperative regulation of C1-domain membrane recruitment polarizes atypical protein kinase C. J. Cell Biol. 222, e202112143 10.1083/jcb.202112143 37589718 PMC10435729

[13] Velnati, S., Centonze, S., Girivetto, F., Capello, D., Biondi, R.M., Bertoni, A. et al. (2021) Identification of key phospholipids that bind and activate atypical PKCs. Biomedicines 9, 45 10.3390/biomedicines9010045 33419210 PMC7825596

[14] Ivey, R.A., Sajan, M.P. and Farese, R.V. (2014) Requirements for pseudosubstrate arginine residues during autoinhibition and phosphatidylinositol 3,4,5-(PO4)3-dependent activation of atypical PKC. J. Biol. Chem. 289, 25021–25030 10.1074/jbc.M114.565671 25035426 PMC4155669

[15] Nakanishi, H., Brewer, K.A. and Exton, J.H. (1993) Activation of the zeta isozyme of protein kinase C by phosphatidylinositol 3,4,5-trisphosphate. J. Biol. Chem. 268, 13–16 10.1016/S0021-9258(18)54107-7 8380153

[16] Palmer, R.H., Dekker, L.V., Woscholski, R., Good, J.A.L., Gigg, R. and Parker, P.J. (1995) Activation of PRK1 by phosphatidylinositol 4,5-bisphosphate and phosphatidylinositol 3,4,5-trisphosphate: a comparison with protein kinase C isotypes (∗). J. Biol. Chem. 270, 22412–22416 10.1074/jbc.270.38.22412 7673228

[17] Lopez-Garcia, L.A., Schulze, J.O., Fröhner, W., Zhang, H., Süß, E., Weber, N. et al. (2011) Allosteric regulation of protein kinase PKCζ by the N-terminal C1 domain and small compounds to the PIF-pocket. Chem. Biol. 18, 1463–1473 10.1016/j.chembiol.2011.08.010 22118680

[18] Tobias, I.S., Kaulich, M., Kim, P.K., Simon, N., Jacinto, E., Dowdy, S.F. et al. (2016) Protein kinase Cζ exhibits constitutive phosphorylation and phosphatidylinositol-3,4,5-triphosphate-independent regulation. Biochem. J. 473, 509–523 10.1042/BJ20151013 26635352 PMC4888060

[19] Mosior, M. and McLaughlin, S. (1991) Peptides that mimic the pseudosubstrate region of protein kinase C bind to acidic lipids in membranes. Biophys. J. 60, 149–159 10.1016/S0006-3495(91)82038-0 1883933 PMC1260046

[20] Pu, Y., Peach, M.L., Garfield, S.H., Wincovitch, S., Marquez, V.E. and Blumberg, P.M. (2006) Effects on ligand interaction and membrane translocation of the positively charged arginine residues situated along the C1 domain binding cleft in the atypical protein kinase C isoforms *. J. Biol. Chem. 281, 33773–33788 10.1074/jbc.M606560200 16950780

[21] Perander, M., Bjørkøy, G. and Johansen, T. (2001) Nuclear import and export signals enable rapid nucleocytoplasmic shuttling of the atypical protein kinase C λ. J. Biol. Chem. 276, 13015–13024 10.1074/jbc.M010356200 11115515

[22] Ways, D.K., Cook, P.P., Webster, C. and Parker, P.J. (1992) Effect of phorbol esters on protein kinase C-zeta. J. Biol. Chem. 267, 4799–4805 10.1016/S0021-9258(18)42903-1 1537859

[23] Johnson, J.E., Giorgione, J. and Newton, A.C. (2000) The C1 and C2 domains of protein kinase C are independent membrane targeting modules, with specificity for phosphatidylserine conferred by the C1 domain. Biochemistry 39, 11360–11369 10.1021/bi000902c 10985781

[24] Katti, S.S., Krieger, I.V., Ann, J., Lee, J., Sacchettini, J.C. and Igumenova, T.I. (2022) Structural anatomy of protein kinase C C1 domain interactions with diacylglycerol and other agonists. Nat. Commun. 13, 2695 10.1038/s41467-022-30389-2 35577811 PMC9110374

[25] Geer, L.Y., Domrachev, M., Lipman, D.J. and Bryant, S.H. (2002) CDART: protein homology by domain architecture. Genome Res. 12, 1619–1623 10.1101/gr.278202 12368255 PMC187533

[26] Kim, H.-Y., Huang, B.X. and Spector, A.A. (2014) Phosphatidylserine in the brain: metabolism and function. Prog. Lipid Res. 56, 1–18 10.1016/j.plipres.2014.06.002 24992464 PMC4258547

[27] Zhang, H., Neimanis, S., Lopez-Garcia, L.A., Arencibia, J.M., Amon, S., Stroba, A. et al. (2014) Molecular mechanism of regulation of the atypical protein kinase C by N-terminal domains and an allosteric small compound. Chem. Biol. 21, 754–765 10.1016/j.chembiol.2014.04.007 24836908

[28] Elbediwy, A., Zhang, Y., Cobbaut, M., Riou, P., Tan, R.S., Roberts, S.K. et al. (2019) The Rho family GEF FARP2 is activated by aPKCι to control tight junction formation and polarity. J. Cell Sci. 132, jcs223743 10.1242/jcs.223743 30872454 PMC6503954

[29] Linch, M., Sanz-Garcia, M., Soriano, E., Zhang, Y., Riou, P., Rosse, C. et al. (2013) A cancer-associated mutation in atypical protein kinase Cι occurs in a substrate-specific recruitment motif. Sci. Signal. 6, ra82 10.1126/scisignal.2004068 24045153

[30] Jumper, J., Evans, R., Pritzel, A., Green, T., Figurnov, M., Ronneberger, O. et al. (2021) Highly accurate protein structure prediction with AlphaFold. Nature 596, 583–589 10.1038/s41586-021-03819-2 34265844 PMC8371605

[31] Graybill, C., Wee, B., Atwood, S.X. and Prehoda, K.E. (2012) Partitioning-defective protein 6 (Par-6) activates atypical protein kinase C (aPKC) by pseudosubstrate displacement. J. Biol. Chem. 287, 21003–21011 10.1074/jbc.M112.360495 22544755 PMC3375524

[32] Kajimoto, T., Caliman, A.D., Tobias, I.S., Okada, T., Pilo, C.A., Van, A.-A.N. et al. (2019) Activation of atypical protein kinase C by sphingosine 1-phosphate revealed by an aPKC-specific activity reporter. Sci. Signal. 12, eaat6662 10.1126/scisignal.aat6662 30600259 PMC6657501

[33] Wang, Y., Seibenhener, M.L., Vandenplas, M.L. and Wooten, M.W. (1999) Atypical PKC ζ is activated by ceramide, resulting in coactivation of NF-κb/JNK kinase and cell survival. J. Neurosci. Res. 55, 293–302 10.1002/(SICI)1097-4547(19990201)55:3<293::AID-JNR4>3.0.CO;2-9 10348660

[34] Wang, G., Krishnamurthy, K., Umapathy, N.S., Verin, A.D. and Bieberich, E. (2009) The carboxyl-terminal domain of atypical protein kinase Cζ binds to ceramide and regulates junction formation in epithelial cells*. J. Biol. Chem. 284, 14469–14475 10.1074/jbc.M808909200 19304661 PMC2682895

[35] Le Good, J.A., Ziegler, W.H., Parekh, D.B., Alessi, D.R., Cohen, P. and Parker, P.J. (1998) Protein kinase C isotypes controlled by phosphoinositide 3-kinase through the protein kinase PDK1. Science 281, 2042–2045 10.1126/science.281.5385.2042 9748166

[36] Cameron, A.J.M., Escribano, C., Saurin, A.T., Kostelecky, B. and Parker, P.J. (2009) PKC maturation is promoted by nucleotide pocket occupation independently of intrinsic kinase activity. Nat. Struct. Mol. Biol. 16, 624–630 10.1038/nsmb.1606 19465915

[37] Soriano, E.V., Ivanova, M.E., Fletcher, G., Riou, P., Knowles, P.P., Barnouin, K. et al. (2016) aPKC inhibition by Par3 CR3 flanking regions controls substrate access and underpins apical-junctional polarization. Dev. Cell 38, 384–398 10.1016/j.devcel.2016.07.018 27554858 PMC4998004

[38] Yamanaka, T., Horikoshi, Y., Sugiyama, Y., Ishiyama, C., Suzuki, A., Hirose, T. et al. (2003) Mammalian Lgl forms a protein complex with PAR-6 and aPKC independently of PAR-3 to regulate epithelial cell polarity. Curr. Biol. 13, 734–743 10.1016/S0960-9822(03)00244-6 12725730

[39] Nunbhakdi-Craig, V., Machleidt, T., Ogris, E., Bellotto, D., White, III, C.L. and Sontag, E. (2002) Protein phosphatase 2A associates with and regulates atypical PKC and the epithelial tight junction complex. J. Cell Biol. 158, 967–978 10.1083/jcb.200206114 12196510 PMC2173154

[40] Kostelecky, B.D. (2009) An Investigation of PKC Isoform Functional Specificity, UCL (University College London), London. Doctoral thesis https://discovery.ucl.ac.uk/id/eprint/18705/

[41] Hornbeck, P.V., Zhang, B., Murray, B., Kornhauser, J.M., Latham, V. and Skrzypek, E. (2015) Phosphositeplus, 2014: mutations, PTMs and recalibrations. Nucleic Acids Res. 43, D512–D520 10.1093/nar/gku1267 25514926 PMC4383998

[42] Rodriguez, J., Peglion, F., Martin, J., Hubatsch, L., Reich, J., Hirani, N. et al. (2017) aPKC cycles between functionally distinct PAR protein assemblies to drive cell polarity. Dev. Cell 42, 400–415.e9 10.1016/j.devcel.2017.07.007 28781174 PMC5563072

[43] Packer, J., Gubieda, A.G., Brooks, A., Deutz, L.N., Squires, I., Ellison, S. et al. (2023) Atypical protein kinase C promotes its own asymmetric localisation by phosphorylating Cdc42 in polarising cells. BioRxiv 10.1101/2023.10.27.563985

[44] DeVries, T.A., Neville, M.C. and Reyland, M.E. (2002) Nuclear import of PKCδ is required for apoptosis: identification of a novel nuclear import sequence. EMBO J. 21, 6050–6060 10.1093/emboj/cdf606 12426377 PMC137198

[45] Seidl, S., Braun, U.B. and Leitges, M. (2012) Functional comparison of protein domains within aPKCs involved in nucleocytoplasmic shuttling. Biology Open 1, 436–445 10.1242/bio.2012505 23213435 PMC3507206

[46] Scotti, M.L., Bamlet, W.R., Smyrk, T.C., Fields, A.P. and Murray, N.R. (2010) Protein kinase C iota is required for pancreatic cancer cell transformed growth and tumorigenesis. Cancer Res. 70, 2064–2074 10.1158/0008-5472.CAN-09-2684 20179210 PMC2881466

[47] Parker, P.J., Justilien, V., Riou, P., Linch, M. and Fields, A.P. (2014) Atypical protein kinase Cι as a human oncogene and therapeutic target. Biochem. Pharmacol. 88, 1–11 10.1016/j.bcp.2013.10.023 24231509 PMC3944347

[48] Wooten, M.W., Vandenplas, M.L., Seibenhener, M.L., Geetha, T. and Diaz-Meco, M.T. (2001) Nerve growth factor stimulates multisite tyrosine phosphorylation and activation of the atypical protein kinase C's via a src kinase pathway. Mol. Cell. Biol. 21, 8414–8427 10.1128/MCB.21.24.8414-8427.2001 11713277 PMC100005

